# Soft Corns Cured by Wiesbaden

**Published:** 1887-12

**Authors:** E. W. Berridge

**Affiliations:** London


### SOFT CORNS CURED BY WIESBADEN.

E. W. BERRIDGE, M. D., LONDON.

1886, December 19th, Miss B., aged about twenty-one, had been suffering for four years from a soft corn between fourth and fifth toes of right foot; the corn shoots and burns; there is also dull aching in outer side of right ankle extending up to hip. She has consulted five allopathic doctors but can get no relief. A year ago an abscess formed between the toes, which removed the corn, but in two months it was as bad as ever. The allopaths tell us to imitate nature in her methods of healing, but they forget that the efforts of nature in such cases are the efforts of a diseased organism, and therefore imperfect.

*Diognosis of the remedy.* The only remedy I could find under "soft corns" was *Wiesbaden*, which has also "burning like fire in feet." I gave *Wiesbaden*$^{200}$ (Jenichen), a dose every other day for fourteen days.

1887. January 1st.—Pain in corn was better in two days; has had it occasionally since, but never so bad as before. Aching in leg quite gone.

January 13th.—No pain for a week; corn began to come away two days ago.

January 25th.—No more pain; corn coming away, leaving a hole.

April 8th.—No more pain; corn has departed, and the hole filled up.

June 10th.—Has remained quite well.

